# Prediction of Incident Diabetes in the Jackson Heart Study Using High-Dimensional Machine Learning

**DOI:** 10.1371/journal.pone.0163942

**Published:** 2016-10-11

**Authors:** Ramon Casanova, Santiago Saldana, Sean L. Simpson, Mary E. Lacy, Angela R. Subauste, Chad Blackshear, Lynne Wagenknecht, Alain G. Bertoni

**Affiliations:** 1 Department of Biostatistical Sciences, Wake Forest School of Medicine, Winston-Salem, North Carolina, United States of America; 2 Department of Epidemiology, Brown University School of Public Health, Providence, Rhode Island, United States of America; 3 Division of Endocrinology and Department of Medicine, University of Mississippi Medical Center, Jackson, Mississippi, United States of America; 4 Department of Epidemiology and Prevention, Wake Forest School of Medicine, Winston-Salem, North Carolina, United States of America; GERMANY

## Abstract

Statistical models to predict incident diabetes are often based on limited variables. Here we pursued two main goals: 1) investigate the relative performance of a machine learning method such as Random Forests (RF) for detecting incident diabetes in a high-dimensional setting defined by a large set of observational data, and 2) uncover potential predictors of diabetes. The Jackson Heart Study collected data at baseline and in two follow-up visits from 5,301 African Americans. We excluded those with baseline diabetes and no follow-up, leaving 3,633 individuals for analyses. Over a mean 8-year follow-up, 584 participants developed diabetes. The full RF model evaluated 93 variables including demographic, anthropometric, blood biomarker, medical history, and echocardiogram data. We also used RF metrics of variable importance to rank variables according to their contribution to diabetes prediction. We implemented other models based on logistic regression and RF where features were preselected. The RF full model performance was similar (AUC = 0.82) to those more parsimonious models. The top-ranked variables according to RF included hemoglobin A1C_,_ fasting plasma glucose, waist circumference, adiponectin, c-reactive protein, triglycerides, leptin, left ventricular mass, high-density lipoprotein cholesterol, and aldosterone. This work shows the potential of RF for incident diabetes prediction while dealing with high-dimensional data.

## Introduction

Type 2 diabetes mellitus (T2DM) has been linked to increased risk of cardiovascular and renal disease, dementia, and cognitive decline [1–3]. This poses a great challenge to the US healthcare system because T2DM and its complications are prevalent and costly. The development of accurate methods for prediction of incident diabetes could facilitate the identification of individuals at high risk of T2DM and the design of prevention strategies. There are many known predictors of T2DM; risk prediction models provide a way to incorporate these risk factors into algorithms that assess an individual’s risk of developing T2DM over a specified period of time [4,5]. Most previous T2DM prediction research is based on traditional statistics, specifically, multivariable regression models that contain a limited set of variables previously identified by clinicians and existing literature as risk factors for T2DM.

Machine learning methods are drawing increasing attention in the area of diabetes detection and risk assessment. They operate in a different manner than traditional approaches described above due to their capabilities to deal successfully with large numbers of variables while producing powerful predictive models. Some machine learning methods have embedded variable selection mechanisms which can detect complex relationships in the data, and thus enable capturing subtle multivariate relationships and nonlinearities that are otherwise difficult to detect.

Support vector machines (SVM) and k-nearest classifiers were used by Farran and colleagues to assess risk of diabetes and its comorbidities in Kuwait[6]. SVM and artificial neural networks were used by Choi and colleagues for pre-diabetes screening in a Korean population[7]. They reported that both approaches outperformed conventional logistic regression in this context. Yu et al. used SVM to detect incident diabetes using data from the National Health and Nutrition Examination Survey[8]. Most of this previous work is based on models that used a reduced set of variables.

Here we used Random Forests (RF) [9] to predict incident diabetes using a large panel of nearly 100 variables. RF is a powerful machine learning method for classification and regression which is based on ensemble learning. A set of “learners” (e.g. classifiers, etc.) are estimated from the data, which are then used to make a decision about assigning a label to a new sample not seen during the estimation process. Some strengths of the RF approach are: 1) it does not over fit the data; 2) it is robust to noise; 3) it has an internal mechanism to estimate error rates; 4) it provides indices of variable importance; 5) it naturally works with mixes of continuous and categorical variables; and 6) it can be used for data imputation and cluster analysis. These properties have made RF increasingly popular, especially in imaging and genetics applications [10–17].

In this work we pursue two main goals. First, we investigate the potential of machine learning methods such as RF for accurate prediction of incident diabetes in a high-dimensional setting defined by a large number of predictors. We hypothesize that RF will compare well to a conventional method such as logistic regression when predicting incident diabetes based on a standard panel of metrics–accuracy, sensitivity, specificity and area under the curve. Second, we aim to identify previously unknown or less investigated predictors of diabetes. To study these questions, we took advantage of the unique opportunity provided by our access to a rich clinical research database collected by the Jackson Heart Study, in a well-characterized African American population known to be more vulnerable to diabetes.

## Methods

### Jackson Heart Study

The Jackson Heart Study (JHS) is a single-site, prospective cohort study of the risk factors and causes of chronic disease in African American adults. JHS was initiated based on the disproportionate burden of chronic disease observed among African Americans in Mississippi, especially within the Atherosclerosis Risk in Communities (ARIC) study site in Jackson [18]. Written informed consent was obtained from participants, and IRBs at University of Mississippi Medical Center and the Wake Forest School of Medicine approved this research.

Participants from the ARIC study were recruited into JHS, and comprise approximately 22% of JHS participants [18,19]. The remaining JHS participants were drawn from a probability sample of African Americans, 21 to 84 years of age, residing in the three counties surrounding Jackson [19]. A total of 5,301 participants were enrolled in JHS at the baseline visit (2000–2004). Study visits included a physical examination, anthropometric measurements, a survey of medical history and cardiovascular risk factors, and collection of blood and urine for biomarker assessment. Visits 2 and 3 were conducted from 2005–2008 and 2009–2013, respectively, at which time diabetes was identified. Diabetes was defined as current use of insulin or oral antidiabetic agent or self-report of physician’s diagnosis, fasting glucose ≥ 126 mg/dl, or hemoglobin A1c ≥ 6.5%. Annual follow-up interviews and cohort surveillance are ongoing. Further details of the study design have been published elsewhere [19,20].

### Random Forests

RF is one of the so-called ensemble methods for classification, because a set of classifiers (instead of one) is generated and each one casts a vote for the predicted label of a given instance provided to the model. Each classifier is a tree built using the classification and regression trees methodology (CART)[21]. In constructing the ensemble of trees, RF uses two types of randomness: first, each tree is grown using a bootstrapped version of the training data. A second level of randomness is added when growing the tree by selecting a random sample of predictors at each node to choose the best split. The number of predictors selected at each node and the number of trees in the ensemble are the two main parameters of the RF algorithm.

The RF developers have reported [9] that the method requires little tuning of the parameters and the default values often produce good results for many problems. Once the forest is built, assigning a new instance to a class is accomplished by combining the trees, using a majority vote. As a result of using a bootstrap sampling of the training data, around one-third of the samples are omitted when building each tree. These are the so-called out-of-the-bag (OOB) samples, which can be used to assess the performance of the classifier and to build measures of importance. In the present report, we used the Gini index to assess variable importance. The Gini index provides a measure of how well a given variable partitions the data during tree construction.

### Statistical analyses

A total of 93 variables from data collected on demographics, anthropometrics, blood biomarkers, medical history, echocardiograms, lifestyle behaviors and socio-economic status were included in the RF approach. We selected these variables: 1) to illustrate RF performance when dealing with a high-dimensional biomedical problem combining continuous and categorical traits, and 2) to uncover potentially unknown predictors of diabetes. Variable selection was also guided by biological plausibility and was limited to variables with less than 5% missing data. These variables are described in S1 and S2 Tables of the supplementary materials. We compared RF models based on these 93 variables (RF^93^), with a logistic regression (LR) model based on the same 93 variables (LR^93^) and a LR model previously published by the ARIC study (LR^ARIC^) [22]. Risk factors considered for incident diabetes prediction in the LR^ARIC^ model included age, race (African Americans vs whites), waist circumference, height, parent history of type 2 diabetes, systolic blood pressure, HDL cholesterol, triglycerides and fasting glucose [22]. We added Hemoglobin A1c not available in ARIC and removed race since all JHS participants are African Americans. These variables are a subset of the variables evaluated by RF^93^. In addition, we trained two-stage versions of both RF and LR where RF and LR models (RF^15^ and LR^15^) were informed by the top 15 ranked features. The two stages models were estimated using training data only.

**Fig 1:**
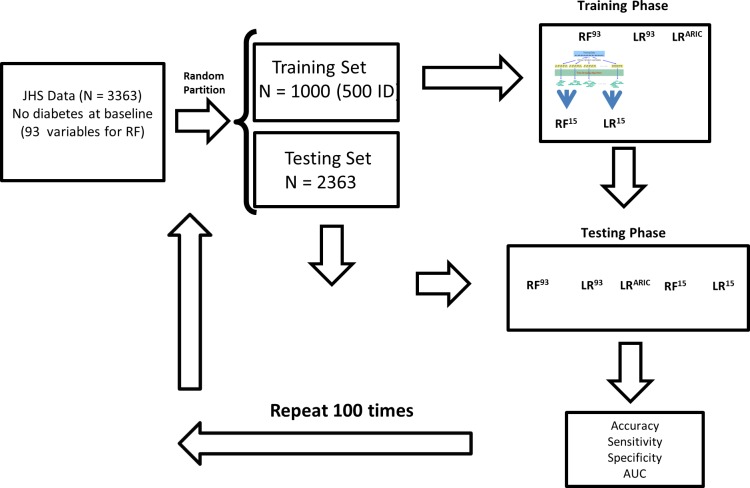
Scheme illustrating the computation experiment designed to compare Random Forests and logistic regression methods.

To estimate the performance of the five models, we partitioned the dataset 100 times into training and testing balanced datasets to deal with an unbalanced classification problem. For each instance, the training dataset included 500 participants who developed diabetes during follow-up (incident diabetes group) and 500 who did not. The remaining data comprised the testing dataset (Fig 1). To avoid possible bias due to differences in dynamic ranges, all predictors were standardized by subtracting the mean and dividing by the standard deviation. Missing data were imputed using the median values of the available data, and as mentioned above, variables missing more than 5% were not considered for selection. The RF and LR models were estimated for the training sets; we used the testing sets to evaluate performance based on accuracy, sensitivity, specificity and area under the curve (AUC). The Gini index produced by RF^93^ was used to rank the importance of the variables in the model. We used the randomForest package in R [23] and its default parameters for RF which are number of trees equal to 500 and number of variables analyzed at each node to find the best split $mtry=\sqrt{p}$ where *p* is the total number of variables in the problem (93 in our case). Finally, although our main results are based on a sample size of 1000 (500 participants per group) we investigated the dependence of performance on sample size for the five models.

## Results

**Fig 2:**
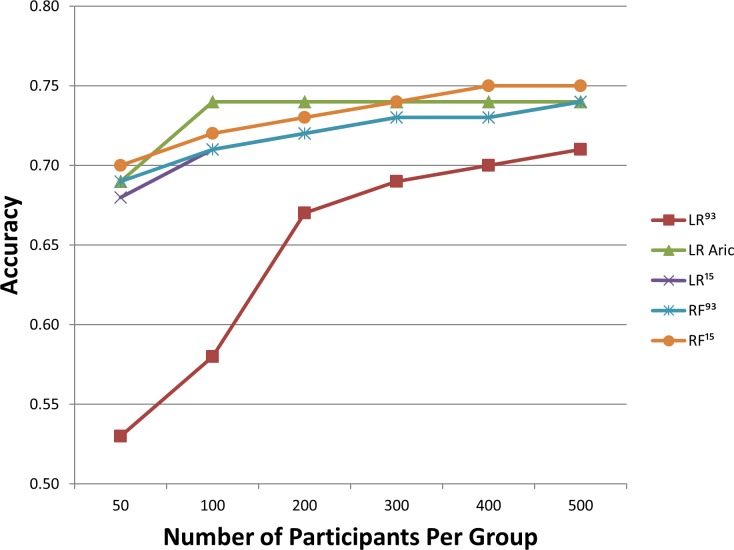
The dependence of classification accuracy on sample size is presented.

Of the 5,301 participants at baseline, 3,363 were at-risk for developing diabetes after excluding those with prevalent diabetes or unconfirmed diabetes status. These remaining participants had an average age of 53.4 years and 63.5% were female (Table 1). Of those at risk, 584 developed incident diabetes during the 9-year follow-up period. Fig 2 shows the relative performance of RF and LR across 100 repetitions of the computations. RF^93^ produced mean values of 74%, 75%, 74% and 0.82 of classification accuracy, sensitivity, specificity and AUC, respectively (Table 2). LR^ARIC^ analyses produced mean values of 74%, 74%, 75% and 0.82 of the same 4 metrics while LR^93^ produced 71%, 70%, 71% and 0.78. The two-stage versions of RF and LR informed by the top 15 variables according to RF rank during training generated little or no gains in performance. Fig 2 shows the dependence of each model on sample size. In general all models performed similarly with the increase of sample size with the exception of LR^93^ which did poorly for small sample sizes but it improved with increasing sample size. RF had longer computation times of 7.93±0.93 seconds vs LR 0.25±0.03 seconds across 100 iterations. RF^15^ and LR^15^ dropped computation times to 6.61±0.47 seconds and 0.03±0.02 seconds respectively.

**Table 1:** Baseline Characteristics by Incident Diabetes Mellitus Status in Prediction of Incident Diabetes in the Jackson Heart Study Cohort using Random Forests.

Baseline Characteristic	Diabetes*(N = 584)	No Diabetes (N = 2779)	All(N = 3363)
Sex			
Male (%)	37.0	36.3	36.5
Female (%)	63.0	63.7	63.5
Age, y	55.2 (11.0)	53.0 (12.8)	53.4 (12.5)
Education			
< High school (%)	19.9	14.4	15.4
High school graduate (%)	18.7	17.7	17.9
Some college (%)	29.6	29.7	29.7
≥ Bachelor’s degree (%)	31.8	38.2	37.1
BMI (kg/m^2^)			
BMI <18.5 (underweight) (%)	0.7	0.2	0.6
BMI 18.5–24.9 (normal weight) (%)	17.0	6.4	15.1
BMI 25–29.9 (overweight) (%)	36.4	26.2	34.6
BMI ≥ 30.0 (obese) (%)	46.0	67.3	49.7
Waist circumference (cm)	105.0(14.1)	97.3(15.6)	98.6(15.6)

*Developed after baseline measurements. Abbreviations: BMI, body mass index.

**Table 2:** Prediction performance of the five models when using sample size 1000 (500 participants per group). The values in each cell correspond to mean and standard deviation across the 100 computations.

Method	Accuracy (%)	Sensitivity (%)	Specificity (%)	AUC
RF^93^	74 (0.02)	75 (0.05)	74 (0.02)	0.82 (0.02)
LR^ARIC^	74 (0.01)	74 (0.05)	75 (0.01)	0.82 (0.02)
LR^93^	71 (0.01)	70 (0.05)	71 (0.01)	0.78 (0.03)
RF^15^	75 (0.02)	74 (0.05)	75 (0.01)	0.82 (0.02)
LR^15^	74 (0.01)	74 (0.04)	74 (0.01)	0.82 (0.02)

RF93 = RF using as input all 93 variables; LR^93^ = logistic regression using as input all 93 variables; LR^ARIC^ = the logistic ARIC model; RF^15^ –two stage RF; LR^15^ = two stage LR.

Table 3 lists the top 15 ranked variables according to the Gini index, one of the RF measures of variable importance. Hemoglobin A1c and fasting plasma glucose were the two most important variables for classification, according to the Gini index. Among the top-ranked variables, RF identified five well-known predictors of T2DM (hemoglobin A1c, fasting plasma glucose levels, waist circumference, triglycerides concentration, and age), predictors that were also part of the LR^ARIC^ model. Several variables not in the ARIC prediction model also ranked high in this study including adiponectin, C-reactive protein, leptin, and aldosterone. S3 Table in the supplementary materials shows which features appear in LR^ARIC^ and RF^15^. The two models have six predictors in common: age, hemoglobin A1c, fasting glucose, waist circumference, HDL cholesterol and triglycerides. Additionally, LR^ARIC^ includes the following predictors that did not make it into RF^15^: African American race, parent history of diabetes, systolic blood pressure and height. African American race was not applicable in this population as all JHS participants are African American. Parent history of diabetes is associated with a wide range of metabolic abnormalities and is strongly associated with development of type 2 diabetes [24]. While the exact mechanisms for this increased risk are not fully understood, it is likely mediated, in part, by genetic as well as shared environmental components among family members. Given the wide array of candidate predictors included as the starting point of the RF^15^ model it’s possible that some of these mechanisms of increased risk associated with family history were captured in other predictors that are in the final RF^15^ model in place of family history of diabetes. Systolic blood pressure was also included in LR^ARIC^ but not in RF^15^. The link between hypertension and diabetes is very well established in the literature so this was surprising to us, however, it is possible that the associated between hypertension and diabetes is mediated through some of the predictors that entered into RF^15^ in place of systolic blood pressure[25]. Finally, height was included in LR^ARIC^ but not in RF^15^. This is likely because BMI was included in RF^15^ and the two measures are typically significantly correlated (albeit inversely).

**Table 3:** Top 15 Variables Found in Random Forest Analyses, according to the Gini Index (N = 1000).

Variable	Gini Index	Diabetes^a^	No Diabetes	p-value*
Hemoglobin A1c (%)	57.4	5.9(0.4)	5.4 (0.4)	< .0001
Fasting plasma glucose (mg/dL)	39.9	97.1 (10.7)	88.8 (7.8)	< .0001
Waist circumference (cm)	19.4	105.0 (14.1)	97.3 (15.6)	< .0001
Adiponectin (ng/mL)	19.0	4091.9 (2750.3)	5566.3 (4032.8)	< .0001
Body mass index (kg/m^2^)	17.6	33.56 (7.0)	30.7 (6.9)	< .0001
High sensitivity C-reactive protein (mg/dL)	15.4	0.6 (0.9)	0.4(0.7)	< .0001
Triglycerides (mg/dL)	14.9	113.88 (59.0)	94.8 (54.7)	< .0001
Age (years)	13.5	55.2 (11.1)	53.0 (12.8)	0.0001
Leptin (ng/mL)	13.2	32.1(27.2)	26.0 (21.9)	< .0001
Body Surface Area (m^2^)	12.6	2.1 (0.2)	2.0 (0.2)	< .0001
eGFR (mL/min/1.73 m^2^)	12.0	85.8 (17.8)	87.2 (16.1)	0.02
2D calculated left ventricular mass (grams)	11.6	157.1 (89.3)	141.8 (39.3)	< .0001
Fasting HDL Cholesterol Level (mg/dL)	11.5	49.3 (12.9)	52.9 (14.8)	< .0001
Fasting LDL Cholesterol Level (mg/dL)	11.2	129.2 (37.9)	127.1 (35.9)	0.15
Aldosterone (ng/mL)	11.0	6.43 (6.48)	5.28 (4.05)	< .0001

* Mean, standard deviations and p-values resulting from Wilcoxon- Mann-Whitney tests.

^a^ Developed after baseline measurements.

An ad hoc analysis (not presented) showed that the prediction is driven by the two top biomarkers (hemoglobin A1c and fasting glucose) while the rest had little or no impact in prediction performance. However most of the top predictors ranked high by RF were statistically significant between the two groups and several of them are factors traditionally or more recently linked to diabetes risk. This suggests that RF is capturing complex interactions present in the data.

## Discussion

Rather than conducting a strict mathematical comparison of these methods, here we have focused on contrasting two approaches to disease risk assessment and statistical modelling. On the one hand are the traditional methods which are parsimonious and based on strong input from experts (e.g. the logistic regression model used in ARIC—LR^ARIC^). On the other hand, there are high-dimensional machine learning approaches represented here by RF that can deal with large number of variables and contain embedded mechanisms for variable importance detection which replaces the experts input during the model building process. Here we used RF to predict incident diabetes based on data from a well-characterized and large clinical research database, the Jackson Heart Study. The full RF^93^ model showed similar prediction performance when compared to a traditional statistical model when predicting incident diabetes in the JHS cohort. The RF model informed by RF top ranked features produced marginal gains in terms of classification accuracy. However, LR^ARIC^, LR^15^ and RF^93^ produced the same AUC suggesting that in our analyses RF was relatively robust to the number of predictors. In addition, our investigation of dependence of performance on sample size that the full RF^93^ model performed better across all sample sizes when compared to LR^93^, illustrating an advantage of machine learning methods over classical statistical methods like LR. LR model based on all variables is not able to deal with high-dimensional data especially when sample sizes are small. However, machine learning (or regularized) versions of LR[26] have proven to be very successful in dealing with problems of much larger dimensionality (number of variables)[27,28]. RF with all variables included was able not only to perform well but also to detect automatically most of the variables in the LR^ARIC^ model with no feedback from experts.

Furthermore, our RF analyses also offered useful information about the potential impact of other, less well-investigated biomarkers not included in the ARIC model; these included adiponectin, C-reactive protein, and leptin. Several studies have shown that higher adiponectin levels are associated with a lower risk of T2DM across diverse populations, consistent with a dose-response relationship[29]. This idea is consistent with the observed values of adiponectin in the JHS cohort at baseline, which were higher for participants who did not develop T2DM during follow-up compared to those who did (see S2 Table). Leptin, a protein secreted by adipose tissue, correlates positively with fat mass and is involved in regulating energy expenditure and insulin sensitivity. Consistent with previous findings suggesting leptin resistance in obese states, JHS participants who developed T2DM during follow-up had, on average, higher levels of leptin at baseline. C-reactive protein is an inflammatory biomarker, minor elevations of which have been reported as a marker of cardiovascular risk in patients with T2DM mellitus [30,31]. In the JHS cohort, those who eventually developed diabetes have been shown to have higher C-reactive protein levels at baseline[32]. In this cohort individuals with higher left ventricular mass are at increased risk of diabetes. Obesity has been linked to an increase in left ventricular mass independent of blood pressure [33]. It is not clear if left ventricular hypertrophy, independent of obesity, increases the risk of T2DM. An interesting finding is the strong association of triglycerides and HDL to the development of T2DM in African Americans. Dyslipidemia of insulin resistance is characterized by elevated triglycerides and low HDL. The universality of this concept has been questioned for African Americans given that this population usually has normal triglycerides levels. The findings in the JHS study suggest that the association of triglyceride levels in the development of T2DM hold in African Americans but the cut off seems to be significantly lower when compared to that of non-Hispanic white populations[34–36].

This study also suggests a potential role for aldosterone as a risk factor in the development of T2DM in African Americans. A relationship between mineralocorticoid receptor activation and decreased insulin sensitivity has been demonstrated both in human studies [37,38]. Furthermore there is some evidence suggesting that mineralocorticoid blockade has the potential of improving insulin resistance. From our study it is unclear if the effect of aldosterone is dependent of the upstream regulator renin. This variable had to be excluded from the analysis due to the number of participants with missing levels.

Our results compare well with other reports of machine learning methods in the literature (see Table 4), especially considering our relatively smaller sample size. We provide more robust performance estimates than those given in the literature since ours are based on taking averages over 100 different partitions of the data into training and testing sets to account for variability in the data. Previously, RF has been used to predict incident diabetes in studies based on electronic health records [39,40]; it showed overall superior performance when compared to other classifiers. Mani et al ([39]) using RF reported results comparable to our study. However, the present study differs from theirs in several respects: 1) They predicted incident diabetes using data from controls and cases six months and one year in advance of disease onset. By contrast, we used JHS data to make longer term predictions (up to 9 years), a more difficult problem; 2) Our estimates of metrics of performance are more robust, since they are based on testing data never seen during estimation and median values over 100 repetitions; and 3) We used a much larger set of predictors, allowing us to evaluate the value of other biomarkers not available in clinical databases. Anderson et al (30) studied a high-dimensional input space based on 298 features. Although some variables were similar to the ones we used, there also were important differences. For example, they used medication information (150 variables), which we did not. On the other hand, we used echocardiographic data and other variables not used in their approach. Although they reported a slightly better performance in their best model, it was based on sample size two to three times larger than ours. Our investigation shows that the performance of the RF approaches improve with sample size. Unlike both previous studies, we used RF measures of variable importance to investigate their relative value for prediction of incident diabetes. In addition, a unique feature of our work is that we focused on a vulnerable African American population using well-characterized clinical data from the JHS. Another work by Guo using a more sophisticated approach based on combination of RF and gradient boosting reported accuracy of 87% when predicting incident diabetes. But they predicted incident diabetes 1 year before disease onset and also a much larger same size (9948).

**Table 4:** Studies investigating prediction of diabetes using machine learning methods.

Reference	Method	Predictors	Sample Size	Type of prediction	Performance
Yu et al. 2010	SVM	family history, age, gender, race and ethnicity, weight, height, waist circumference, BMI, hypertension, physical activity, smoking, alcohol use, education, and household income(NHANES Cohort).	4915	Cross-sectional	AUC = 0.73
Mani et al. 2012	RF	A1c,Sys BP,Diastolic BP, GLU, BMI, Creatinine, HDL, MDRD, Triglycerides, Race, Gender, Age(EHR Data).	2280	1 year ahead	AUC = 0.80
Choi et al. 2014	SVMANN	age, body mass index, hypertension, gender, daily alcohol intake, and waist circumference(KNHANES cohort)	4685	Cross-sectional	AUC = 0.74
Anderson et al. 2016		age,gender,systolic/diastolic BP, Height, Wieght, BMI, 150 ICD9 code, 150 common meds(HER data).	9948	Cross-sectional	AUC = 0.81
Luo 2016	BRT + RF	The data set includes information ondemographics, diagnoses, allergies, immunizations, lab results, medications, smoking status, and vital signs.	9948	1 year ahead	Accuracy = 87.4%
Our Study	RF^15^	Hemoglobin A1c, fasting glucose, waist circumference, adiponectin, BMI, hs-CRP, triglycerides, age, leptin, body surface area, eGFR, 2D calculated left ventricular mass, HFL cholesterol, LDL cholesterol, aldosterone.	3633	8 years ahead	AUC = 0.82Accuracy = 75%

ANN–Artificial Neural Networks; BRT +RF–Combination of Boosting Regression Trees and RF classifiers.

Based on our results, RF methods have utility in the health care setting, where large datasets with thousands of well-characterized phenotypes and large numbers of participants are common. Furthermore, development of biomedical technologies will very likely lead to cheaper data acquisition in the future, making available even more biomarkers that could be included in mathematical models to make predictions about health outcomes.

Our study is not without limitations. We did not test other available traditional models or other high-dimensional machine learning approaches. We did not validate our model using other datasets. In the two-stage approach we did not optimize the number of top ranked features to be included in the second step of the two-stage procedure. We selected adhoc the top 15 produced by RF^93^. Some of the biomarkers ranked in the top 15 by RF were correlated (e.g. BMI and waist circumference) which should be taken into account when interpreting these results. In general the two approaches to modeling (traditional and machine learning) should be seen as complementary rather than exclusive. For example, an approach such as RF can be used for hypothesis setting via pattern discovery while more traditional methods like LR can be used for further hypothesis testing. Even though during model building phase RF needed very little input from experts, ultimately the results of any mathematical model, including data mining methods, need to be validated by experts.

## Conclusion

In summary, this work shows the potential of high-dimensional machine learning analyses for prediction of incident diabetes. RF was evaluated using data from the JHS to predict incident diabetes in a well characterized cohort of African Americans followed for 8 years. Even though a large body of research has accumulated to develop methods to predict incident diabetes most of the published work is based on traditional statistical methods. Machine learning approaches are beginning to gain the attention of the community and within this context our work is an additional contribution to the field that characterized performance of RF in a high-dimensional setting where many biomarkers not usually included in traditional models were evaluated. We believe that in general our results compare well with other reports in the literature but more work remains to be done to increase the quality of prediction. Machine learning technologies can be used to develop powerful predictive models of incident diabetes with relatively little input from human experts in the model- building phase. Methods such as RF have internal mechanisms that allow the detection of influential variables on prediction performance which are at the core of the pattern detection paradigm embodied by the datamining approaches. Future work will seek to validate these results in other large databases, increase the sample size to improve performance or deploy more sophisticated modeling approaches.

## Supporting Information

S1 Table Description of Variables Used to Predict Incident Diabetes in Random Forests Analyses. (DOCX)

S2 Table Baseline Values (mean ± standard deviation) of Continuous Variables Used to Predict Incident Diabetes in Random Forests Analyses. (DOCX)

S3 Table Degree of coincidence between LR^ARIC^ and RF^15^. (DOCX)
