# Supplementary material for: Prediction of Incident Diabetes in the Jackson Heart Study Using High-Dimensional Machine Learning
Source: PLoS One. 2016 Oct 11;11(10):e0163942. doi: 10.1371/journal.pone.0163942 (PMC5058485; doi:10.1371/journal.pone.0163942)
Supplement: S1 Table — (DOCX) [file pone.0163942.s001.docx]

**Table S1.** Description of Variables Used to Predict Incident Diabetes in Random Forests Analyses

| Variable | Definition |
| --- | --- |
| currentSmoker | Indication of participant's current cigarette smoking status |
| everSmoker | Indication of whether the participant has ever smoked cigarettes |
| CVDHx | Cardiovascular Disease History |
| parent_diabetes | Parental Diabetes History (Yes / No) |
| age | Age in Years |
| height | Height (cm) |
| waist | Waist Circumference (cm) |
| BMI | Body Mass Index (kg/m^2) |
| sbp | Systolic Blood Pressure (mmHg) |
| dbp | Diastolic Blood Pressure (mmHg) |
| BPjnc7 | JNC 7 BP Classification ( Normal, Pre-HTN, Stage I, Stage II ) |
| HTN | Hypertension Status (Yes / No) |
| HbA1c | NGSP Hemoglobin A1c (%) |
| FPG | Fasting Plasma Glucose Level (mg/dL) |
| ldl | Fasting LDL Cholesterol Level (mg/dL) |
| hdl | Fasting HDL Cholesterol Level (mg/dL) |
| trigs | Fasting Triglyceride Level (mg/dL) |
| LEPTIN | Concentration of Leptin (Plasma ng/mL) |
| HSCRP | High Sensitivity C-Reactive Protein (Plasma mg/dL) |
| ENDOTHELIN | Endothelin-1 (Plasma pg/mL) |
| ALDOSTERONE | Concentration of Aldosterone (Plasma ng/dL) |
| sCort | Concentration of Cortisol Levels (Plasma ug/dL) |
| adiponectin | Concentration of adiponectin (Plasma ng/mL) |
| SCr | CC Calibrated Serum Creatinine (mg/dL) |
| eGFR | eGFR MDRD (mL/min/1.73 m^2^) |
| DialysisEver | Self-reported dialysis |
| MajorScarPost | ECG determined Posterior Major Scar |
| MinorScarPost | ECG determined Posterior Minor Scar |
| RepolarPost | ECG determined Posterior Repolarization |
| MIpost | ECG determined Posterior MI |
| MajorScarAntLat | ECG determined Anterolateral Major Scar |
| MinorScarAntLat | ECG determined Anterolateral Minor Scar |
| RepolarAntLat | ECG determined Anterolateral Repolarization |
| MIecg | ECG determined MI (Yes, No) |
| Afib | Atrial Fibrillation |
| cystatinC | Concentration of cystatin C (Serum mg/L) |
| ECHA13 | Body Surface Area (m^2^) |
| ECHA16 | LA dilation (0=None 1= Mild 2=Mod 3=Severe 9=Missing) |
| ECHA17 | LV dilation (0=None 1= Mild 2=Mod 3=Severe 9=Missing) |
| ECHA18 | RA dilation (0=None 1= Mild 2=Mod 3=Severe 9=Missing) |
| ECHA19 | RV dilation (0=None 1= Mild 2=Mod 3=Severe 9=Missing) |
| ECHA20 | Aortic dilation (0=None 1= Mild 2=Mod 3=Severe 9=Missing) |
| ECHA21 | LV Hypertrophy (Qualitative Assessment ) (0=None 1= Mild 2=Mod 3=Severe 9=Missing) |
| ECHA22 | Pericardial effusion (0=None 1= Small 2=Mod 3=Large 9=Missing) |
| ECHA23 | LV ejection fraction (Semi quantitative;nearest 5%) |
| ECHA24 | LV regional wall motion (N=Normal B=Border line A=Abnormal 9=Missing) |
| ECHA25 | Aortic leaflets (0=Normal 1=Sclerosis 2,3,4=Mild/Mod/Severe Stenosis 9=Missing) |
| ECHA26 | Mitral leaflets (0=Normal 1=Sclerosis 2,3,4=Mild/Mod/Severe Stenosis 9=Missing) |
| ECHA27 | Mitral regurgitation (0=Normal 1=Sclerosis 2,3,4=Mild/Mod/Severe 9=Missing) |
| ECHA28 | Aortic regurgitation (0=Normal 1=Sclerosis 2,3,4=Mild/Mod/Severe 9=Missing) |
| ECHA29 | Tricuspid regurgitation (0=Normal 1=Sclerosis 2,3,4=Mild/Mod/Severe 9=Missing) |
| ECHA31 | Mitral Anular Calcification (0=None 1=Mild 2=marked 9=Missing) |
| ECHA32 | Mitral Valve Prolapse (0=None 1=Mild 2=marked 9=Missing) |
| ECHA50 | 2D diastolic IV septum thickness in millimeters |
| ECHA52 | 2D diastolic LV diameter in millimeters |
| ECHA53 | 2D systolic LV diameter in millimeters |
| ECHA54 | 2D diastolic posterior wall thickness in millimeters |
| ECHA56 | 2D LA diameter (end-systole) in millimeters |
| ECHA57 | 2D aortic root diameter (end-diastole) at sinuses in millimeters |
| ECHA58 | 2D calculated left ventricular mass (grams) |
| HCAA1 | Do you have a particular place for health assistance? |
| MSRA32 | Past 2 weeks, take any aspirin..? |
| MSRA35A | Now taking other non-steroidal... drugs? |
| MSRA36A | Have you used vinegar? |
| MSRA37A | Past 2 weeks have you used epsom salts? |
| MSRA38A | Lemon juice or lemon? |
| MSRA39A | Past 2 weeks have you used garlic? |
| MSRA40A | Past 2 weeks did you use Teas? |
| MSRA41A | Past 2 weeks did you use Roots? |
| MSRA42A | Any other home remedies used (2 weeks)? |
| MSRA43A | Any other home remedies? |
| HCAA4 | Have you seen a dentist in the past 12 months? |
| HCAA5 | What was the last time you had a routine physical? |
| HCAA6 | How hard has it been for you to get health services |
| HCAA7 | Do you have private health insurance coverage? |
| HCAA8 | Medicaid/public aid coverage |
| HCAA9 | Medicare coverage |
| HCAA11 | Satisfied w/doctor |
| PDSA27A | Receive income from investments in past year? |
| PDSA27B | Receive income from Social security in past year? |
| PDSA27C | Receive Worker's compensation in past year? |
| PDSA27D | Receive Unemployment compensation in past year? |
| PDSA27E | Receive ADC or AFDC in past year? |
| PDSA27F | Receive Food stamps in past year? |
| PDSA27G | Receive other welfare program in past year? |
| PDSA27H | Receive Supplemental security income in past year |
| PDSA27I | Income from gambling in past year? |
| SOCA2 | Currently living with spouse/another person in relationship |
| SOCA5 | How many close friends can you talk to? |
| SOCA6 | How many relatives are you close to? |
| SOCA7 | Number of friends/relatives you see once per month |
| SOCA1A | Married/Single/Divorced/Widowed |
| SOCA8A | Do you belong to social/church groups? |

ECG - electrocardiographic.
