# Supplementary material for: Prediction of Incident Diabetes in the Jackson Heart Study Using High-Dimensional Machine Learning
Source: PLoS One. 2016 Oct 11;11(10):e0163942. doi: 10.1371/journal.pone.0163942 (PMC5058485; doi:10.1371/journal.pone.0163942)
Supplement: S2 Table — (DOCX) [file pone.0163942.s002.docx]

**Table S2.** Baseline Values (mean ± standard deviation) of Continuous Variables Used to Predict Incident Diabetes in Random Forests Analyses

| Variable | Diabetes (N = 584) | No Diabetes (N = 2779) | Probt |
| --- | --- | --- | --- |
| ALDOSTERONE | 6.43 (6.48) | 5.28 (4.05) | <.0001 |
| BMI | 33.56 (7.02) | 30.69 (6.93) | <.0001 |
| ECHA13 | 2.06 (0.24) | 1.98 (0.24) | <.0001 |
| ECHA50 | 9.37 (3.47) | 8.8 (1.48) | <.0001 |
| ECHA52 | 48.61 (4.47) | 48.23 (4.21) | 0.2186 |
| ECHA53 | 29.7 (4.85) | 29.7 (4.4) | 0.5343 |
| ECHA54 | 8.79 (1.96) | 8.38 (1.56) | <.0001 |
| ECHA56 | 35.62 (4.2) | 34.73 (4.26) | <.0001 |
| ECHA57 | 31.75 (3.18) | 31.58 (3.18) | 0.2728 |
| ECHA58 | 157.06 (89.27) | 141.81 (39.34) | <.0001 |
| ENDOTHELIN | 1.4 (0.65) | 1.3 (0.55) | 0.0001 |
| FPG | 97.05 (10.65) | 88.81 (7.81) | <.0001 |
| HSCRP | 0.61 (0.9) | 0.44 (0.66) | <.0001 |
| HBA1C | 5.88 (0.39) | 5.42 (0.44) | <.0001 |
| LEPTIN | 32.14 (27.22) | 26.01 (21.88) | <.0001 |
| RENIN | 1.83 (7.86) | 1.06 (3.19) | 0.0034 |
| SCR | 1.03 (0.44) | 1.01 (0.36) | 0.1103 |
| ADIPONECTIN | 4091.86 (2750.29) | 5566.28 (4032.75) | <.0001 |
| AGE | 55.17 (11.02) | 53.01 (12.76) | 0.0002 |
| CYSTATINC | 0.76 (0.31) | 0.71 (0.31) | <.0001 |
| DBP | 80.38 (10.19) | 79.07 (10.43) | 0.0054 |
| EGFR | 85.81 (17.75) | 87.23 (16.1) | 0.0212 |
| HDL | 49.25 (12.87) | 52.87 (14.81) | <.0001 |
| HEIGHT | 169.13 (9.29) | 169.21 (9.34) | 0.8286 |
| LDL | 129.15 (37.94) | 127.14 (35.89) | 0.1526 |
| SCORT | 9.48 (3.81) | 9.46 (3.92) | 0.6255 |
| SBP | 127.99 (17.88) | 124.35 (17.34) | <.0001 |
| TRIGS | 113.88 (58.98) | 94.84 (54.72) | <.0001 |
| WAIST | 104.89 (14.12) | 97.26 (15.61) | <.0001 |

*p-values corresponding to a Wilcoxon rank sum test are included.
