# Supplementary material for: Prediction of Incident Diabetes in the Jackson Heart Study Using High-Dimensional Machine Learning
Source: PLoS One. 2016 Oct 11;11(10):e0163942. doi: 10.1371/journal.pone.0163942 (PMC5058485; doi:10.1371/journal.pone.0163942)
Supplement: S3 Table — (DOCX) [file pone.0163942.s003.docx]

**Table S3.** Degree of coincidence between LR^ARIC^ and RF^15^.

|  | LR^ARIC^ | RF^15^ |
| --- | --- | --- |
| Age | X | X |
| Fasting glucose | X | X |
| Waist circumference | X | X |
| HDL cholesterol | X | X |
| Triglycerides | X | X |
| Parent history of diabetes | X |  |
| SBP | X |  |
| Height | X |  |
| A1C |  | X |
| Adiponectin |  | X |
| BMI |  | X |
| Hs-CRP |  | X |
| Leptin |  | X |
| Body surface area |  | X |
| eGFR |  | X |
| 2D calculated left ventricular mass |  | X |
| LDL cholesterol |  | X |
| Aldosterone |  | X |
